# An international collaborative study to determine the prevalence of contagious caprine pleuropneumonia by monoclonal antibody-based cELISA

**DOI:** 10.1186/1746-6148-10-48

**Published:** 2014-02-24

**Authors:** Armelle Peyraud, François Poumarat, Florence Tardy, Lucía Manso-Silván, Karomatullo Hamroev, Tillo Tilloev, Mullojon Amirbekov, Karim Tounkara, Charles Bodjo, Hezron Wesonga, Isabel Gacheri Nkando, Shiferaw Jenberie, Martha Yami, Eric Cardinale, Deodass Meenowa, Mahmad Reshad Jaumally, Tahir Yaqub, Muhammad Zubair Shabbir, Nadia Mukhtar, Mohibullah Halimi, Ghulam Mohammad Ziay, Willy Schauwers, Hafizullah Noori, Ali Madad Rajabi, Stéphane Ostrowski, François Thiaucourt

**Affiliations:** 1 Centre de coopération internationale en recherche agronomique pour le développement (CIRAD) UMR CMAEE, Montpellier F-34398, France; 2 INRA, UMR1309 CMAEE, Montpellier F-34398, France; 3 ANSES, Laboratoire de Lyon, UMR Mycoplasmoses des Ruminants, 31 Avenue Tony Garnier, Cedex 07, Lyon F-69364, France; 4 Université de Lyon, VetAgro, 1 av Bourgelat Marcy l”étoile, Lyon 69280, France; 5 State Veterinary Inspection Services, Ministry of Agriculture, Rudaki Av 44, Dushanbe 734025, Tajikistan; 6 Pan African Veterinary Vaccine Center (AU-PANVAC), P.O. Box 1746, Debre-Zeit, Ethiopia; 7 Kenya Agricultural Research Institute (KARI), Veterinary Research Centre, KARI Muguga North, P.O. Box 32–00902, Kikuyu, Kenya; 8 National Veterinary Institute, P.O. Box 19, Debre-Zeit, Ethiopia; 9 CIRAD UMR CMAEE, Centre de Recherche et de Veille de l”Océan Indien, Sainte-Clotilde 97491 Réunion, France; 10 Ministère de l”Agriculture, de la technologie alimentaire et des ressources naturelles, A. de Plevitz street Long Mountain Réduit, Maurice, France; 11 Quality Operations Laboratory, University of Veterinary and Animal Sciences, Lahore 54600, Pakistan; 12 Animal Health and Production, Ministry of Agriculture, Jamal Mina, Kabul, Afghanistan; 13 Animal Health and Development Program, Phase II (EU project), Ministry of Agriculture, Jamal Mina, Kabul, Afghanistan; 14 Wildlife Conservation Society, Afghanistan Program, Kabul, Afghanistan; 15 Wildlife Conservation Society, New York, USA

**Keywords:** Contagious caprine pleuropneumonia, Competitive ELISA, Seroprevalence, Kenya, Ethiopia, Mauritius, Tajikistan, Afghanistan, Pakistan, Vaccine quality control

## Abstract

**Background:**

Few serological tests are available for detecting antibodies against *Mycoplasma capricolum* subsp. *capripneumoniae*, the causal agent of contagious caprine pleuropneumonia (CCPP). The complement fixation test, the test prescribed for international trade purposes, uses a crude antigen that cross-reacts with all the other mycoplasma species of the “mycoides cluster” frequently infecting goat herds. The lack of a more specific test has been a real obstacle to the evaluation of the prevalence and economic impact of CCPP worldwide. A new competitive ELISA kit for CCPP, based on a previous blocking ELISA, was formatted at CIRAD and used to evaluate the prevalence of CCPP in some regions of Kenya, Ethiopia, Mauritius, Tajikistan and Pakistan in an international collaborative study.

**Results:**

The strict specificity of the test was confirmed in CCPP-free goat herds exposed to other mycoplasma species of the “mycoides cluster”. Prevalence studies were performed across the enzootic range of the disease in Africa and Asia. Seroprevalence was estimated at 14.6% in the Afar region of Ethiopia, whereas all the herds presented for CCPP vaccination in Kenya tested positive (individual seroprevalence varied from 6 to 90% within each herd). In Mauritius, where CCPP emerged in 2009, nine of 62 herds tested positive. In Central Asia, where the disease was confirmed only recently, no positive animals were detected in the Wakhan District of Afghanistan or across the border in neighboring areas of Tajikistan, whereas seroprevalence varied between 2.7% and 44.2% in the other districts investigated and in northern Pakistan. The test was also used to monitor seroconversion in vaccinated animals.

**Conclusions:**

This newly formatted CCPP cELISA kit has retained the high specificity of the original kit. It can therefore be used to evaluate the prevalence of CCPP in countries or regions without vaccination programs. It could also be used to monitor the efficacy of vaccination campaigns as high-quality vaccines induce high rates of seroconversion.

## Background

Contagious caprine pleuropneumonia (CCPP) has been known as a clinical condition for 140 years [1], but the burden and distribution of this disease remain largely unknown. The causal agent of CCPP was first isolated in 1976 [2], but it was not given its species name until 1993: *M. capricolum* subsp. *capripneumoniae* (Mccp) [3]. Conspicuous macroscopic lesions are observed in animals with acute disease. These lesions are restricted to the pleural cavity and consist of unilateral pleuropneumonia with profuse pleural effusion, but diagnosis may still be difficult. Until the development of specific PCR assays [4,5], it was difficult to obtain laboratory confirmation of CCPP. Autopsies are rarely performed on goats dying from the disease and, when samples are sent to the laboratory for isolation of the causal agent, other mycoplasmas, such as *M. mycoides* subsp. *capri* (Mmc), *M. capricolum* subsp. *capricolum* (Mcc) and *M. ovipneumoniae,* are often isolated. This is due to the extremely fastidious growth of Mccp, which is overgrown by faster growing mycoplasmas. Furthermore, animals are often treated with antibiotics, hampering isolation of the causal agent. Despite great improvements in the formulation of culture media, the isolation of Mccp remains very difficult [6]. It was thought that the use of PCR for the molecular detection of Mccp would greatly facilitate the diagnosis of CCPP and provide more accurate information about the prevalence of the disease.

However, there have been very few declarations of CCPP outbreaks to the OIE in the last 15 years, due to a lack of awareness of this disease and possible confusion with other diseases, such as “peste des petits ruminants” (PPR) or *Pasteurella* infections. Little is known about the economic impact of CCPP, although participatory epidemiological surveillance, without the need for laboratory confirmation, may prove a useful approach. For example, Turkana pastoralists in Kenya rank CCPP as one of the principal diseases affecting their goats, together with PPR and sarcoptic mange [7]. The impact of CCPP and the potential threat represented by this disease have been greatly underestimated.

Until recently, CCPP was believed to be confined to Africa and the Middle East. Its presence in Asia was confirmed only recently, with the isolation of Mccp strains in China [8] and the detection of Mccp by PCR in Pakistan [9] and Tajikistan. Molecular epidemiology studies have shown that the Mccp strains circulating in Asia belong to a specific clade supported by significant bootstrap values [10]. This indicates that the presence of the disease in Asia is not the result of a recent introduction, which would have resulted in Asian Mccp genotypes closely resembling genotypes found elsewhere. Indeed, it was suggested that CCPP was present in India as early as 1914 [11]. The disease was also recognized in the European part of Turkey in 2005 [12] and poses a threat to the Balkan countries of the European Union. CCPP has been shown to infect wildlife species held in captivity for conservation purposes in Qatar [13] but has also been detected in free-ranging wildlife in Tibet [14]. This is a matter of concern not only for free-ranging endangered species in which CCPP may occur [15], but also for CCPP-free countries importing wild species for propagation purposes or for zoological collections.

CCPP is difficult to control. The vaccines against CCPP consist of inactivated Mccp antigen with saponin as an adjuvant [16]. If effective, these vaccines should induce marked seroconversion, and this need-s to be investigated furher. The antibodies induced by vaccination can interfere with the results of disease prevalence studies, but cELISA could be used to assess the efficacy of vaccination campaigns. Vaccination efficacy has been assessed in experimental trials, leading to the definition of an optimum Mccp antigen concentration of 0.15 mg/dose, which yields a protective immune response [17].

The lack of a reliable, commercially available specific ELISA test for detecting Mccp antibodies was recognized as a key issue during the design of an EU-funded project aiming to control neglected animal diseases in Africa through vaccination (VACNADA). A blocking ELISA based on the use of a specific monoclonal antibody recognizing Mccp was developed in 1994 [18]. This kit has recently been modified to obtain a heat-stable kit produced to quality assurance standards. We describe here the use of this new kit to evaluate the prevalence of CCPP in various countries and to evaluate the seroconversion induced by the CCPP vaccine.

## Methods

### Formatting of the cELISA kit

The same components of the kit developed in 1994 [18] were used, but the kit was modified in collaboration with a private company, IDEXX-Montpellier SAS. The hybridoma cell line “4.52” developed by CIRAD was revivified and subcloned to check its stability. The main objective was then to produce stable precoated plates and to design a protocol yielding results similar to those originally reported, in terms of specificity and sensitivity. Serum samples known to be positive and negative were used to achieve this end.

The final CCPP cELISA kit protocol differed slightly from the original published protocol, as it was a strict competitive assay rather than a blocking assay. We minimized the variability of incubation time by first mixing diluted sera (1/10) with a fixed quantity of monoclonal antibody in a normal non-coated plate and then transferring the mixture to a precoated plate and incubating at 37°C for one hour, with gentle shaking. The plates were then incubated for 30 minutes with the conjugate and 20 minutes with the tetramethyl-benzidine substrate. Plates were washed manually between steps in the protocol. The final protocol for the CCPP cELISA very closely resembled that used for contagious bovine pleuropneumonia testing (IDEXX-Montpellier 309 SAS, ref: P05410-10).

Once the kit had been developed, a validation batch was produced by IDEXX-Montpellier SAS (“beta kit” CIRAD-Vacnada) and subjected to accelerated stability testing by incubation at 37°C for 2 months, with testing at various time points.

In this competitive ELISA, the final results are given by the formula [(ODMab-ODTest)/(ODMab-ODconjugatecontrol)]*100 and are expressed as the “percentage inhibition” (PI).

Whenever possible, the kit was validated according to the rules laid down in chapter 1.1.5 of the OIE manual of standards (http://www.oie.int/en/international-standard-setting/terrestrial-manual/access-online/).

### Determination of the cutoff point maximizing diagnostic specificity

This cutoff point was established by analyzing 478 serum samples from 16 goat herds from France, which is a CCPP-free country. The status of each herd, in terms of the circulation of mycoplasmas from the “Mycoides cluster”, was determined before the study, by culturing various samples, as previously described [19]. For each herd, a bulk milk sample was collected, together with individual milk. Seven herds were considered to be mycoplasma-infected, because *M. mycoides* subsp. *capri* (Mmc) or *M. capricolum* subsp. *capricolum* (Mcc) strains were isolated from the bulk tank milk and/or from at least one individual milk sample.

### Quality assurance during testing

Once the cutoff point had been established, the confidence interval around this value was established in the CIRAD laboratory, taking several factors into account. First, an “internal reference material” (IRM) serum with a titer slightly higher than the cutoff point was produced and freeze-dried. This IRM serum was tested on four different occasions, by three technicians and at various positions in the plates, with the generation of 392 results in total.

All the ELISA tests were conducted in accordance with the general guidelines of ISO17025 at CIRAD-UMR15, which has obtained accreditation from the French Committee for Accreditation for this cELISA test (COFRAC certificate N° 1–2207).

### Seroprevalence in Central Asia

**Figure 1:**
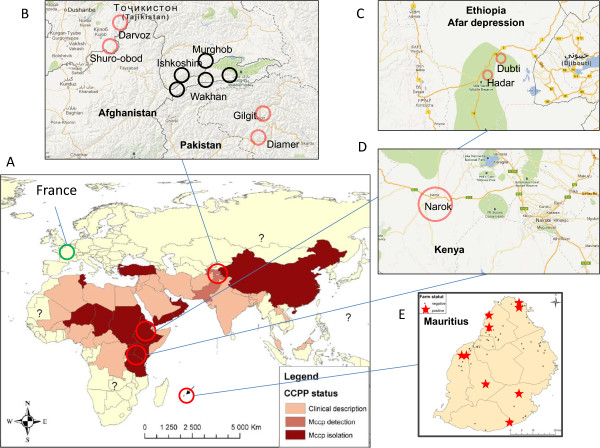
**Map showing the countries and regions studied in the CCPP-cELISA serological survey. A**: World map showing the various locations. The green circle represents France, where the specificity of the test was validated, **B**: Central-Asian location around the Wakhan district in Afghanistan, **C**: Afar depression in Ethiopia, **D**: Narok district in Kenya, **E**: Reunion Island; the dots indicate the locations of the sampled herds that tested negative by CCPP cELISA, whereas the red starts indicate the locations of the sampled herds testing positive.

The cELISA was used to investigate the presence of CCPP in remote districts of Afghanistan, Pakistan and Tajikistan centered on the Pamir mountain range. In each country, two (Pakistan) or three (Afghanistan, Tajikistan) sampling areas at least 50 km apart were selected, to ensure broad geographical coverage (Figure 1). In Afghanistan, 359 goats from eight sites in the district of Wakhan, Badakhshan Province were selected. No clinical cases of CCPP had been reported in these districts (Ostrowski pers. obs. 2012). In Pakistan, 410 goats from six sites in the Diamer and Gilgit districts, Gilgit-Baltistan Province were sampled. Clinical cases of CCPP were reported, but Mccp was never isolated (Ghulam Abbas pers. comm. 2012) at these sites. In Tajikistan, 395 goats from eight sites, including 196 from the Shuro-obod District, Khatlon Province and Darvoz District, Gorno Badakhshan Autonomous Oblast (Province) were sampled. Clinical cases of CCPP occurred in 2008 and Mccp was detected at these sites in 2010 [20] In addition, 199 goats from the Ishkoshim and Murghob districts in Gorno Badakhshan Autonomous Oblast, in which no clinical cases of CCPP were reported, were also sampled.

### Seroprevalence in infected countries in East Africa and Mauritius

Serum samples were collected from Kenya, in the area around Narok, 300 km west of Nairobi, where CCPP is known to be enzootic. Herds were selected on the basis of the willingness of their owners to participate in a vaccination campaign organized by the VACNADA project. There was no evidence of clinical signs of CCPP in the herds at the time of sampling. All animals from the selected herds were sampled (10 herds, N = 895). In Ethiopia, samples were obtained from the Afar region, in which CCPP is known to have been present since its introduction in 1990, in the eastern part of the country [21]. One thousand serum samples were collected from the Dubti and Hadar districts, from two sites per district in which no vaccination had been performed during the last five years. We sampled 75% of the herds, by selecting at random at least 20 serum samples per herd, from animals that were at least two months old.

In addition, serum samples were also collected from Mauritius, where CCPP has recently been identified [22]. Sixty-two herds were selected to constitute a sample representative of the whole island and 10 to 15 adult goats were randomly selected from each herd (62 herds, *N* = 720).

### Seroconversion in vaccinated animals

A reference batch of CCPP vaccine was produced by CIRAD, as described by Rurangirwa [16], but with slight modification of the published protocol, including inactivation before freeze-drying. Briefly, the Mccp antigen consisted of a four-day culture of the “Abomsa” strain. The mycoplasmas were concentrated and washed three times by centrifugation at 12000 × *g* for 15 minutes and washing with PBS. The concentrated suspension (1 mg/ml) was inactivated by the binary ethylenimine procedure [23,24] and freeze-dried. The inactivation process was checked by culture. The protein content of each vial was determined by the bicinchoninic acid method, with bovine serum albumin as the reference. The saponin adjuvant was prepared immediately before use, by dissolving saponin powder (Sigma, ref. S4521, batch 010 M7015) to obtain the desired amount per dose.

Six-year old naive goats were immunized at CIRAD with a vaccine meeting OIE requirements: 0.15 mg of antigen and 3 mg of saponin per dose. The immunization protocol was approved by the local ethics committee and the goats were housed in an animal facility controlled by French veterinary services (certificate: C34-172-20). Serum samples were collected at weekly intervals for two months and then after five months. Various combinations of antigen and saponin doses were then prepared and used to immunize goats at the African Union Pan African Veterinary Vaccine Center (AU-PANVAC, Ethiopia). Saponin-adjuvanted vaccines are registered in Ethiopia and do not require specific authorization for use. The animal facilities at AU-PANVAC are located within the National Veterinary Institute, Debre-Zeit, which is the reference laboratory for vaccine production and quality testing in Ethiopia. We tested the following ratios, expressed in mg mycoplasma protein/mg saponin per dose: A = 0.15/3; B = 0.15/0.3; C = 0.05/3; D = 0.05/0.3; E = 0/3; F = 0.15/0. Sera were collected weekly for six weeks and tested by cELISA.

## Results

### Kit performance

When the kit was stored at 37°C, the values for the control monoclonal antibody (Mab) declined over time. They remained above the accepted limit (0.5 OD) for three weeks, after which, the percentage inhibition (PI) value obtained for the laboratory internal positive reference serum decreased slightly, from a mean of 61PI to 54PI, but clearly remained within acceptable limits.

Once the kit had been formatted and a preliminary batch had been produced in an industrial production process by IDEXX-Montpellier SAS, the specificity of the test was assessed on 478 serum samples of French origin of known infection status. The distribution of results followed a Gaussian curve, with a mean PI of 25.5. There was no correlation between mean cELISA titer and mycoplasma infection status. Indeed, *Mycoplasma mycoides* subsp. *capri* (Mmc) was the most frequently isolated mycoplasma species (six of the seven herds for which infection was detected on the basis of tests on milk), whereas *Mycoplasma capricolum* subsp. *capricolum* (Mcc) was detected only once. The 40 serum samples with the highest titers, including two individual serum samples with PI values of 56 and 57, were retested twice, to determine the titer more precisely. The highest mean PI value for a negative serum was 54. A cutoff point of 55PI was applied, so the test was strictly specific.

An internal reference serum, with a titer close to the cutoff point, was tested in 392 replicates, on four different dates, by three technicians. All the results were pooled and the distribution was evaluated. The curve obtained was strictly Gaussian, with a mean PI value of 56. The standard deviation (SD) of this mean was 3.5. Further testing over a one-year period showed the uncertainty of measurement around the mean to be 8PI for serum samples with titers around the cutoff point. This uncertainty was estimated taking into account all the reagents, equipment and parameters critical for a cELISA test, as specified by the ISO-17025 standard. The most important factors are the kit itself, the precision of the pipettes, the training and experience of the technicians, and the linearity and repeatability of the ELISA reader. This uncertainty of measurement has two main components: the performance of the kit itself and the way the test is implemented in the laboratory. The same kit may therefore yield different uncertainties in different laboratories.

We present here only the data generated at CIRAD. However, the test was also performed locally elsewhere, notably in Ethiopia, Kenya, Mali, Mauritania, Pakistan, Senegal and Tajikistan. At all these sites, correct results were obtained for the controls, demonstrating the robustness of the kit.

Five sites were selected for the evaluation of CCPP seroprevalence with this cELISA (Figure 1-A). France was selected as a representative site at which CCPP is absent but other mycoplasmas of the mycoides cluster are isolated frequently. The other sites selected were in countries in which CCPP is known to occur.

### Seroprevalence in Central Asia

A preliminary test was performed on seven serum samples collected during a CCPP outbreak confirmed by PCR, in South-West Tajikistan. These serum samples were collected from goats that had shown respiratory signs in the previous weeks or months. Six of the seven samples tested positive and titers were very high in some cases, with PI values of up to 90.

The 359 serum samples collected from the Wakhan District in Afghanistan and the 199 serum samples retrieved from the contiguous Ishkoshim and Murghob districts in Tajikistan tested negative. These results are highly consistent with the absence of clinical signs of CCPP in this part of the Central Asian highlands (Figure 1-B).

In the province of Gilgit-Baltistan in northern Pakistan, serological results confirmed the clinical observations suggestive of CCPP in this area, but the results obtained differed between sampling sites. In the Gilgit District only four of the 150 serum samples tested were positive, giving a prevalence of 2.7% (95% CI: 0.7%-6.7%). By contrast, in the Diamer District, 115 of the 260 serum samples tested were positive, giving a prevalence of 44.2% (95% CI: 38.1%-50.5%), a value significantly higher than that obtained for Gilgit. The reasons for this difference are unknown, but further sampling, with the testing of a larger sample spread over a broader geographical area should increase our understanding of the variation of CCPP seroprevalence in Gilgit-Baltistan.

In the Shuro-Obod District and the contiguous Darvoz District in southern Tajikistan, 20 of the 197 serum samples analyzed tested positive, giving a prevalence of 10.1% (95% CI: 6.3%-15.2%), confirming the clinical observations suggestive of CCPP in this region bordering Afghanistan [20]. More precise results should become available in the future, as a nationwide serological survey is currently being carried out as part of a technical cooperation project of the Food and Agriculture Organization (FAO) of the United Nations. This project should provide precise estimates of CCPP prevalence in the Tajik goat stock.

### Seroprevalence in infected African countries and Mauritius

We tested 1000 goat serum samples from the Afar region of Ethiopia, from sites at which vaccination had never been carried out; 146 of these samples tested positive (Figure 1-C). The individual seroprevalence in this region was therefore 14.6% (95% CI: 13%-17%). Prevalence varied between sites, with values of 10 and 12% obtained for the Dubti district and of 5.3 and 23% for the Hadar district.

In the Narok district in Kenya (Figure 1-D), serum samples were collected from each of the goats in 10 herds (46 to 180 serum samples per herd), the owners of which agreed to participate in the CCPP vaccination campaign. Positive results were obtained for every herd tested. Seroprevalence differed considerably between herds, with between 6 and more than 90% of the serum samples from a given herd testing positive. Herd prevalence may therefore be highly heterogeneous in enzootic zones and may depend, in particular, on the time elapsed since the start of the CCPP outbreak in the herd concerned.

In Mauritius, only nine of the 62 herds tested positive, with at least one serum sample having a PI value greater than 63 (the cutoff point plus the uncertainty of measurement) (Figure 1-E). Herd prevalence was therefore estimated at about 15%, with an individual prevalence of 8%. Within the positive herds, the percentage of serum samples testing positive was high, from 26 to 80%. In Mauritius, it was observed that positive herds followed animal husbandry practices favoring contact with goats from other farms.

### Detection of antibodies following vaccination

Three goats vaccinated at CIRAD with a batch of the reference vaccine displayed rapid marked seroconversion, which was detectable after as little as one week. PI values peaked three weeks after vaccination (74, 87 and 92 respectively) and remained stable at for at least five weeks thereafter. The same animals were retested five months after vaccination: two continued to test positive (PI values of 62 and 76, respectively), whereas the third tested negative.

**Figure 2:**
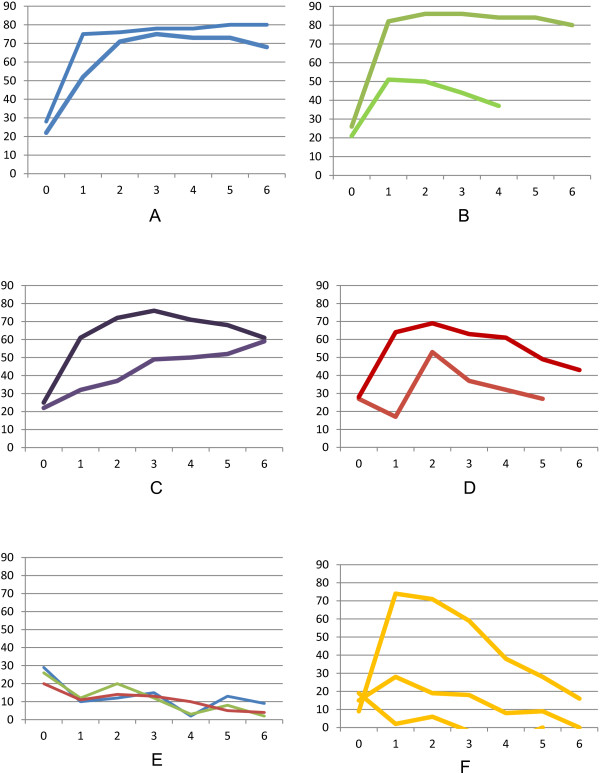
**Seroconversion, as assessed by CCPP cELISA, in goats vaccinated with various amounts of *Mycoplasma capricolum *subsp. *capripneumoniae *antigen and saponin adjuvant.** Each line represents the seroconversion of one animal. The *x* axis corresponds to the time in weeks post vaccination and the *y* axis corresponds to the percentage inhibition values. **A**: Goats vaccinated with the reference vaccine (0.15 mg antigen, 3 mg saponin), **B**: goats vaccinated with 0.15 mg antigen and 0.3 mg saponin, **C**: goats vaccinated with 0.05 mg antigen and 3 mg saponin, **D**: goats vaccinated with 0.05 mg antigen and 0.3 mg saponin, **E**: goats receiving saponin alone (3 mg), **F**: goats receiving antigen alone (0.15 mg).

At AU-PANVAC, groups of two to three goats were vaccinated with various proportions of antigen and adjuvant. Goats vaccinated with the reference vaccine displayed seroconversion of a similar intensity to that seen at CIRAD (Figure 2-A). By contrast, the use of lower concentrations of antigen (Figure 2-C and D) and/or adjuvant (Figure 2-B and D) resulted in seroconversion that was either less pronounced or lasted for a shorter period. The negative controls, which received an injection of saponin alone, displayed no seroconversion (Figure 2-E). Only one of the animals receiving antigen alone (Figure 2-F) displayed transient seroconversion. No seroconversion was observed in the other two. These results confirm that the cELISA was able to detect the antibodies induced by CCPP vaccination. They also show that vaccine quality directly affected the intensity and duration of seroconversion. Lower antigen content and the use of smaller amounts of adjuvant resulted in weaker responses.

## Discussion

This newly formatted CCPP cELISA kit performed significantly better than the previous version. The new version of the kit includes highly heat-stable precoated plates in sealed packaging, and the test performs well after incubation for three weeks at 37°C. An additional advantage of this kit is its modification to use the same protocol as the contagious bovine pleuropneumonia cELISA kit (IDEXX-Montpellier SAS, France). Laboratories that are familiar with the bovine test can therefore easily perform the caprine test.

The very high specificity of the CCPP cELISA, at close to 100%, was similar to that of the previous blocking ELISA. In this study, we did not use goat herds and animals chosen at random from a CCPP-free country. Instead, we deliberately also included goat herds infected with other mycoplasmas of the “mycoides cluster”, to increase the stringency of the test. Such animals would probably have given cross-reactions in the less specific complement fixation test (CFT), which uses a crude antigen [25,26]. This cELISA can be used for the specific detection of CCPP. Another commercially available serological test for CCPP, the latex agglutination test, is based on the use of beads coated with Mccp polysaccharide [27]. This test is highly suitable for the rapid confirmation of outbreaks in the field, as agglutination is mostly triggered by IgM antibodies. However, cELISA is likely to be more suitable for epidemiological surveillance and seroprevalence studies, as it is independent of Ig type. Our choice of a high cutoff point, to maximize the diagnostic specificity of the test, would be expected to decrease diagnostic sensitivity. Further trials will certainly be required to determine the true sensitivity of this test as there is no gold standard serological test. Such analyses could be achieved by the analysis of serum samples from experimentally infected animals. The estimates of prevalence obtained in this study may therefore constitute an underestimate of the true prevalence of CCPP.

The results of cELISA tests confirmed that CCPP was enzootic in the lowlands of Kenya and Ethiopia, with a high prevalence in the herds tested. These findings are consistent with those of participatory enquiries [28], which have shown that goat owners in these semi-arid nomadic lands rank CCPP first among the threats to their herds [7]. In Kenya, the sampled goats originated from herds presented for vaccination. The willingness of the owners to have their animals bled and vaccinated may be linked to previous exposure to CCPP. Our sample cannot therefore be considered to be completely random and the results represent no more than an estimate of the seroprevalence of CCPP.

The epidemiological situation for CCPP in Mauritius differs from that in East Africa. The disease was not introduced into Mauritius until 2009, and it will be interesting to follow the progress of this disease in the coming years and to analyze it in terms of the control strategies that may be implemented.

Results for Central Asia were highly variable and depended on the geographical origin of the samples. These findings highlight the need for widespread serological investigations, to clarify the distribution of the disease across Asia, which is currently poorly understood.

The absence of CCPP in the Wakhan District of North-East Afghanistan may, to some extent, reflect the remoteness of this district, which provides some protection against the introduction of infected animals.

The high seroprevalence observed in East Africa indicates a lack of success of the control strategies implemented in this region, despite the local production of CCPP vaccines. However these vaccines are difficult to produce. This makes them expensive and limits their use in the field.

The widespread presence of mycoplasmas of the “mycoides cluster” around the Mediterranean basin may potentially mask the emergence of CCPP. These mycoplasmas induce a wide range of lesions in affected goats, including CCPP-like pleuropneumonia. Hence, goat owners and veterinary surgeons may not detect CCPP quickly enough to warn local health authorities and curtail the risk of epizootics. The cELISA may, therefore, prove very useful for the development of epidemiological surveillance activities in regions at risk of CCPP introduction, such as the Balkans.

Finally the preliminary results obtained in this study indicate that this serological test could be used to evaluate the quality of CCPP vaccines or vaccination campaigns, by monitoring the seroconversion induced in vaccinated animals. The collection of serum samples before vaccination and then one and two months after vaccination should provide clear evidence of seroconversion if the vaccine was produced according to the OIE standards. Seroconversion indicates that sufficient Mccp antigen and adjuvant were present in the vaccine to induce the proper response. This cELISA could therefore be used as an initial test of potency, such tests currently being absent from the reference quality control protocols. Further trials are required to determine whether there is a strict correlation between antibody levels, as assessed with this cELISA, and protection. Such trials would also be useful to determine the true sensitivity of the test, which is difficult to evaluate with serum samples collected in the field. Quality control is particularly important for vaccines, as the industrial product used is generated on a large scale and may behave differently from the original laboratory product for which efficacy was demonstrated [29,30]. The cELISA test could be used, in particular, to evaluate the stability of the vaccine in liquid form after storage at +4°C.

## Conclusions

The newly formatted CCPP cELISA proved to be highly specific and robust. Localized prevalence studies confirmed that the prevalence of CCPP was high in the lowlands of East Africa, where goat-rearing is a key activity in rural communities. Prevalence studies in central Asia showed that some regions may still be CCPP-free, whereas others are clearly highly infected. These findings could lead to better control strategies, including the use of cELISA for the monitoring of vaccination campaign efficacy. Finally, this cELISA could be used as a surveillance tool in CCPP-free regions at risk of disease introduction, including all the regions bordering infected zones.

### Ethical considerations

Goat serum samples were collected in field studies carried out in various countries without ethics committees (e.g., Tajikistan and Afghanistan). Serum sampling was organized by the national veterinary services in accordance with local legislation. It included only blood sampling from the jugular vein, which is not considered to cause any animal pain.

When CCPP vaccination was performed, notably in Ethiopia, the vaccines used were preparations that had already been authorized for market release in the country concerned, or vaccine preparations with similar constituents but at lower doses.

## Abbreviations

OIE: World Organization for Animal Health; FAO: Food and Agriculture Organization of the United Nations; WHO: World Health Organization; ISO: International Organization for Standardization.

## Competing interests

The authors have no competing interests to declare. IDEXX Montpellier SAS is to produce and market this CCPP cELISA kit in the near future. CIRAD, acting as reference laboratory for CCPP for the OIE, is to control the conformity of every batch produced by IDEXX.

## Authors” contributions

AP developed the new kit, analyzed the serum samples and set up quality management at CIRAD. FTh coordinated the study and drafted the manuscript. FTa, SO, HW, SJ, CB, EC were responsible for selecting study areas and coordinated the serum sampling in France, Central Asia, Kenya, Ethiopia, vaccine control experiments and Mauritius respectively. SO, LMS, FTa, FP, AP provided critical feedback on the manuscript. KH, TT, MA, KT, IGN, MY, DM, MRJ, TY, MZS, NM, MH, GMZ, WS, HN, AMR participated in the study design in their respective countries and gathered epidemiological data on CCPP. All authors read and approved the final manuscript.
